# From scrolling to swinging: a chain mediation model of psychological needs, social media use, participation motivation, and behavioral intention in Chinese college pickleball players

**DOI:** 10.3389/fpsyg.2026.1883121

**Published:** 2026-08-19

**Authors:** Jing Gao, Jianfeng Hu, Chunbao Yang, Xiaohui Dai

**Affiliations:** Yantai Institute of Science and Technology, Yantai, China

**Keywords:** behavioral intention, chain mediation, college students, participation motivation, pickleball, psychological needs, social media use

## Abstract

The present study explores the internal mechanism by which psychological needs are associated with college students’ intention to play pickleball, focusing on the mediating roles of social media use and participation motivation. By integrating three theories—Self-Determination Theory (SDT), the Theory of Planned Behavior (TPB), and Uses and Gratifications Theory (UGT)—this paper aims to construct and test a chain mediation model. A cross-sectional questionnaire survey, based on stratified convenience sampling, was conducted among 1,992 college students from 12 universities in six provinces of China. The questionnaire contained four subscales: psychological needs (12 items), social media use (5 items), participation motivation (18 items), and behavioral intention (10 items). The hypothesized paths were tested with structural equation modeling (maximum-likelihood estimation) and bootstrap mediation analysis (5,000 resamples). In the simultaneous structural model, psychological needs were positively associated with behavioral intention (*β* = 0.205, *p* < 0.001), social media use (*β* = 0.596, *p* < 0.001), and participation motivation (*β* = 0.782, *p* < 0.001); social media use (*β* = 0.419, *p* < 0.001) and participation motivation (*β* = 0.355, *p* < 0.001) were also significant direct predictors of behavioral intention. Both mediators carried significant indirect effects, and the sequential chain path accounted for 5.2% of the total effect. The model explained 76.2% of the variance in behavioral intention. All nine hypotheses were empirically supported. Because diagnostic tests indicated substantial common-method variance, these associations should be interpreted with that qualification in mind. This research advances an integrated theoretical framework linking psychological needs, digital engagement, and sport participation intention, and provides practical implications for sports marketers, campus administrators, and policy makers seeking to promote emerging leisure sports through social media.

## 1 Introduction

Pickleball is a new racket sport that combines elements of tennis, badminton, and table tennis. In recent years, it has achieved leaps and bounds across the globe. Originated in the United States in the mid-1960s, the sport has grown from a niche leisure activity to one of the fastest-growing sports in the world (Sports and Fitness Industry Association, 2023). In China, pickleball has spread rapidly among college students because of its simplicity and accessibility, strong social attributes, and the contemporary health trends. China’s Ministry of Education has increasingly emphasized the diversification of campus sports, and pickleball has become a typical case of the adoption of emerging leisure activities in the field of higher education.

The factors driving college students to participate in pickleball are not pure sports competition, but have important implications for campus sports policy, public health promotion, and sports industry development. College students are the key group in sports participation research, because the sports habits formed at this stage often continue to adulthood (Telama et al., 2005). In addition, this group is digital natives, and social media is deeply embedded in their daily information acquisition and social interaction modes. The intersection of psychological needs, digital media consumption, and sports participation intention constitute a rich theoretical and practical research problem.

This paper integrates three mature theoretical perspectives for analysis. Self-Determination Theory (SDT; Deci and Ryan, 1985) provides a basic perspective for understanding how basic psychological needs such as autonomy, competence, and relatedness drive human behavior. The Theory of Planned Behavior (TPB; Ajzen, 1991) provides a fully validated framework for linking motivational processes and behavioral intentions (Hagger et al., 2002a; Hagger and Hamilton, 2024). The Uses and Gratifications Theory (UGT; Katz et al., 1974) explains why individuals tend to specific media channels to meet specific needs (Kim and Kim, 2020). By weaving these three theoretical clues, this paper aims to build a more comprehensive and powerful interpretation model than any single perspective.

The rationale for combining these three perspectives is that they operate at complementary explanatory levels. SDT specifies the distal motivational engine—the basic psychological needs that energize behavior; UGT describes the channel-level mechanism through which need-related orientations are translated into engagement with need-relevant media content; and TPB models the proximal intention-formation system in which attitudinal and normative forces crystallize into behavioral plans. Prior research supports each pairwise linkage: SDT and TPB have been formally integrated in meta-analytic tests of health-behavior sequences (Hagger and Chatzisarantis, 2009); SDT and UGT have been jointly applied to explain social media use (Sheldon et al., 2011; Gao et al., 2021); and UGT has been fruitfully applied to the short-video platforms central to the present context (Omar and Dequan, 2020; Meng and Leung, 2021). Taken together, the three theories imply an ordered sequence—needs, media engagement, motivation, and intention—that is theoretically coherent and empirically testable.

The novelty of this method lies in the theoretical triangulation. As far as we know, few prior studies have examined psychological needs, social media use, participation motivation, and behavioral intention in an integrated framework.

Against this backdrop, the present study addresses three specific gaps in the existing literature. First, although self-determination theory is widely used in the field of sport and exercise, systematic reviews and meta-analyses have concentrated on established activities such as running, swimming, and gym-based exercise (Teixeira et al., 2012), and its extension to emerging leisure sports such as pickleball remains limited. The growing pickleball literature has so far centered on older adults’ well-being and serious leisure (Heo et al., 2018; Buzzelli and Draper, 2020; Cerezuela et al., 2023) or on intention models grounded in the theory of planned behavior (Wang et al., 2023; Huang et al., 2026), leaving need-based mechanisms among Chinese college students largely unexamined. Second, the mediating role of social media use in translating psychological needs into sport participation intentions has not been systematically investigated: chained mediation from fitness-related social media use to exercise outcomes has been demonstrated for general fitness content (Xiao et al., 2025), but not for emerging racket sports. Third, existing mediation studies typically test simple rather than sequential (chain) mediation configurations and may therefore overlook the mechanisms by which distal variables are associated with behavioral outcomes through ordered, multi-stage processes (cf. Hagger and Chatzisarantis, 2016).

To address these gaps, this paper proposes and empirically tests a chain mediation model in which psychological needs are associated with behavioral intention directly and through two sequential mediators: social media use and participation motivation. In doing so, the study offers three contributions, previewed here and developed in the Discussion: (a) an integrated SDT–UGT–TPB account of sport participation intention in which each theory occupies a complementary explanatory level; (b) an extension of self-determination theory to an emerging leisure sport and to the pre-participation stage; and (c) evidence on the need–media–motivation sequence in a large Chinese college sample.

## 2 Theoretical framework

### 2.1 Self-determination theory and psychological needs

According to Self-Determination Theory, the three basic psychological needs form the basis of intrinsic motivation and mental health: autonomy (the need to experience will and self-approval), competence (the need to feel effective in activities), and relatedness (i.e., the sense of belonging—the need to connect with others and feel cared for) (Deci and Ryan, 2000). When these needs are met, individuals naturally tend to experience need satisfaction. In sports situations, demand satisfaction has been consistently proved to predict autonomous motivation, persistence, and mental health (Ng et al., 2012; Alkasasbeh and Akroush, 2025).

Pickleball seems particularly suited to meet these three basic needs (Ryan and Deci, 2020). The sport provides a variety of forms of participation (singles, doubles, mixed doubles), allowing participants to exercise autonomy by choosing preferred patterns; progressive skill curve provides continuous competency related feedback; the inherent social attributes of doubles cultivate relatedness (Heo et al., 2018). Therefore, this paper expects that college students with strong psychological needs will show stronger intention to participate in pickleball.

### 2.2 Theory of planned behavior

The Theory of Planned Behavior (Ajzen, 1991) identifies three antecedents of behavior intention: behavior attitude, subjective norms, and perceived behavior control. A large number of meta-analysis evidences confirm that TPB construct can explain the significant variance in exercise and exercise intention (Hagger et al., 2002a; Hagger and Hamilton, 2024). The participation motivation in this model reflects the attitude and normative power emphasized by TPB, and is operationalized as the motivation power to guide individuals to participate in the pickleball.

In this paper, participation motivation is positioned as the proximal predictive variable of behavioral intention, mediating the influence of more distal configuration. This sort of ranking conforms to TPB’s hierarchical logic, that is, background factors (in this paper, psychological needs and media exposure) shape specific attitudes and normative beliefs that directly inform the intention formation.

### 2.3 Uses and gratifications theory

The Uses and Gratifications Theory shifts the focus of analysis from what the media do to people to what people do with the media (Katz et al., 1974). The theory proposes that individuals actively choose media channels to meet specific needs, including cognitive, emotional, personal integration, social integration, and tension release. In the contemporary digital environment, social media platform has become the main channel for sports related information discovery, consumption, and sharing (Filieri et al., 2015).

For college students, social media is the primary channel for sports-related content. The short video platform displays pickleball highlights, technical tutorials, and user generated content, which can stimulate initial interest and maintain participation. This paper conceptualizes the use of social media as active participation related to sports, rather than passive or general screen time exposure.

### 2.4 Theoretical integration and hypothesis development

Integrating the three theories yields an ordered process model. Psychological needs (SDT) constitute the distal motivational engine; social media use (UGT) is the channel through which need-related content and participation opportunities are discovered; participation motivation (TPB) is the proximal attitudinal-motivational mechanism; and behavioral intention is the immediate antecedent of actual participation. This ordering implies both direct associations and a chain of indirect effects, from which nine hypotheses are derived. Throughout this paper, “prediction” is used in the statistical sense of the directed associations specified by the theoretical model.

Self-determination theory holds that satisfaction of the needs for autonomy, competence, and relatedness energizes motivated engagement and intentional persistence (Deci and Ryan, 2000). In exercise settings, need satisfaction has been consistently linked to stronger intentions to be physically active: a systematic review of SDT-based exercise studies concluded that need satisfaction is a robust correlate of autonomous motivation and exercise participation (Teixeira et al., 2012), and need-related constructs have been linked to exercise and physical-activity intentions in diverse samples (Wilson and Rodgers, 2004; Hagger et al., 2006; Behzadnia et al., 2018). Because pickleball offers autonomy-supportive participation formats, competence-relevant feedback, and inherently social play, strong basic psychological needs are expected to be associated with stronger intention to participate.

*H1*: Psychological needs positively predict behavioral intention to participate in pickleball.

Uses and gratifications theory implies that engagement with need-relevant media content brings participation opportunities, behavioral models, and social norms into salience. Among pickleball players, behavioral intention has been identified as the key proximal correlate of sustained participation (Wang et al., 2023), and fitness-related social media use has been found to be associated with exercise intention and behavior in Chinese college samples (Xiao et al., 2025). Active engagement with pickleball content—highlights, tutorials, and peer-generated posts—is therefore expected to be associated with stronger participation intention.

*H2*: Social media use positively predicts behavioral intention.

A proposition shared by SDT and UGT is that salient psychological needs orient individuals toward media environments that promise need-relevant gratifications. Need-related states have been shown to predict patterns of Facebook use (Sheldon et al., 2011) and WeChat use intensity (Gao et al., 2021), and gratifications sought—information, entertainment, and social connection—are robust predictors of short-video engagement on TikTok and comparable platforms (Omar and Dequan, 2020; Meng and Leung, 2021). Students with strong autonomy, competence, and relatedness needs are thus expected to turn to social media for pickleball-related content.

*H3*: Psychological needs positively predict social media use.

Within the theory of planned behavior, intention is the immediate antecedent of behavior, and autonomous motives have been shown to predict intention within TPB frameworks (Hagger et al., 2002b; Hagger and Chatzisarantis, 2009). For pickleball specifically, motivational and attitudinal factors have been identified as central correlates of the intention to play (Huang et al., 2026). Where sport-specific evidence is scarce, we follow the reviewers’ suggestion and draw on e-sports and online-gaming analogs: TPB-based studies of e-sports participation consistently report strong motivation–intention associations (Leung et al., 2021; Chung et al., 2022), and SDT-based work shows that e-sports engagement is grounded in basic psychological needs (Qian et al., 2022).

*H4*: Participation motivation positively predicts behavioral intention.

The motivational sequence from needs to contextual motivation is well established in hierarchical models of motivation (Vallerand, 1997). In digital play contexts, need satisfaction has been shown to be associated with sustained motivational engagement (Ryan et al., 2006; Przybylski et al., 2010), and among Chinese college students, autonomy-supportive environments that satisfy basic needs have been linked to autonomous motivation toward leisure-time physical activity (Abula et al., 2020). We therefore expect psychological needs to be strongly associated with participation motivation.

*H5*: Psychological needs positively predict participation motivation.

Media engagement can also feed motivation: exposure to need-congruent sport content supplies behavioral models, skill-relevant information, and social proof that plausibly strengthen the motivation to participate. Fitness-related social media use has been found to be associated with intrinsic motivation in a Chinese college sample (Xiao et al., 2025), and UGT research shows that gratifications obtained from short-video use reinforce continued engagement motives (Omar and Dequan, 2020).

*H6*: Social media use positively predicts participation motivation.

If psychological needs orient students toward social media (H3) and social media use is associated with intention (H2), then social media use should statistically mediate the needs–intention association. Comparable mediated sequences have been documented: need satisfaction has been linked to excessive WeChat use via usage intensity (Gao et al., 2021), and fitness social media use has been shown to carry indirect associations with exercise outcomes (Xiao et al., 2025).

*H7*: Social media use mediates the association between psychological needs and behavioral intention.

The trans-contextual model and related SDT–TPB integrations position autonomous motivation as the mechanism by which need-related experiences are carried forward into intentions (Hagger et al., 2003, 2005; Hagger and Chatzisarantis, 2016). Participation motivation should therefore mediate the needs–intention association in the pickleball context as well.

*H8*: Participation motivation mediates the association between psychological needs and behavioral intention.

Finally, the two mediators may operate sequentially: needs orient students toward social media (H3), media engagement feeds participation motivation (H6), and motivation crystallizes into intention (H4), forming a need → media → motivation → intention chain. Chained indirect paths of this kind have been reported for fitness social media use (Xiao et al., 2025) and in need–media research on general social platforms (Gao et al., 2021).

*H9*: Social media use and participation motivation sequentially (chain) mediate the association between psychological needs and behavioral intention.

## 3 Research model and hypotheses

**Figure 1:**
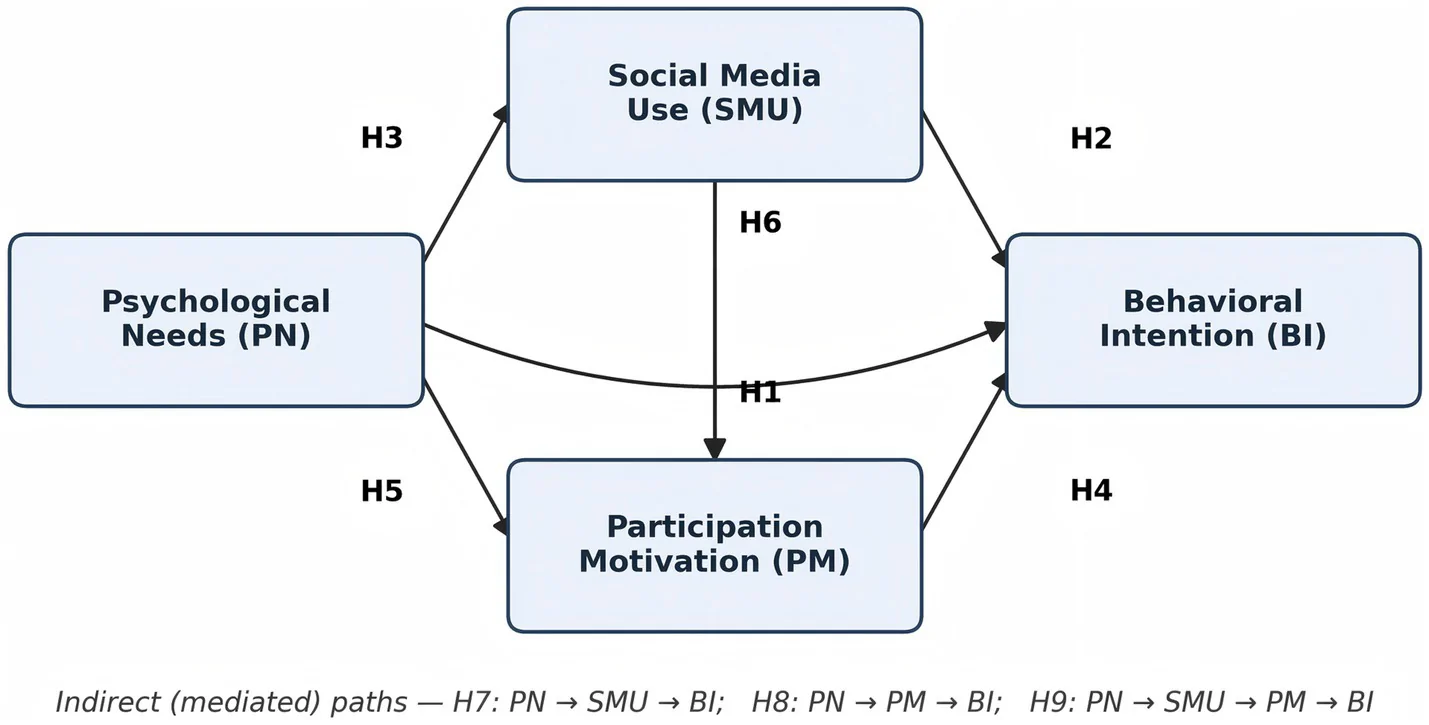
Conceptual research model. PN, psychological needs; SMU, social media use; PM, participation motivation; BI, behavioral intention.

Figure 1 shows the hypothetical research model. Psychological needs are exogenous variables, which affect behavior intention through four paths: direct path (H1), indirect path through social media use (H7), indirect path through participation motivation (H8), and chain path through two sequential mediators (H9). Social media use and participation motivation have additional direct effects on behavioral intention (H2 and H4, respectively). Psychological needs predict two mediating variables (H3 and H5), and social media use also predicts participation motivation (H6), thus completing the chain structure.

The model was specified *a priori* on the basis of the theoretical arguments developed in Section 2.4; all hypothesized paths were fixed before the data were analyzed, and no *post hoc* model modifications were introduced.

## 4 Research methods

### 4.1 Subjects and procedures

Using stratified convenience sampling, with stratification by region (eastern, central, and western China) and institution type, participants were recruited from 12 universities across six provinces. Recruitment was conducted through university sports associations, physical education departments, and social media channels, and data were collected via an online questionnaire platform between March and April 2026. The inclusion criteria were enrollment in a higher education institution and provision of informed consent; participation was voluntary and uncompensated. To reduce common method bias at the procedural level, respondents were assured of the anonymity and confidentiality of their responses, and attention-check items were embedded in the questionnaire to identify careless responding (Podsakoff et al., 2003).

A total of 2,085 responses were returned. Screening removed incomplete questionnaires (*n* = 58) and questionnaires that failed the attention checks (*n* = 34); one further record could not be recovered during data-file preparation, yielding a final analytic sample of *N* = 1,992 students, an effective response rate of 90.82% of the distributed invitations.

An a-priori power analysis for covariance structure models (MacCallum et al., 1996), computed with Soper’s (2026) SEM sample-size calculators, indicated that with d*f* = 939, *α* = 0.05, and power = 0.80, the minimum sample sizes required for tests of close fit (H0: RMSEA ≤ 0.05 versus H1: RMSEA = 0.08) and not-close fit (H0: RMSEA ≥ 0.05 versus H1: RMSEA = 0.01) are approximately 35 and 51, respectively—far below the achieved *N* = 1,992. The sample also comfortably exceeds conventional *N*: item (1,992/45 ≈ 44:1) and *N*: parameter guidelines for structural equation modeling.

### 4.2 Measuring tools

All constructs were measured with validated scales adapted to the pickleball context. The 5-point Likert scale was used to score from 1 (very disagree) to 5 (very agree).

All scales were adapted to the pickleball context through a procedure: a panel of sport-psychology faculty reviewed the adapted items for content validity; a forward–back translation check was performed to verify the semantic equivalence of the Chinese items; and a pilot test with students from one university led to minor wording refinements before the main data collection.

The 12-item scale adapted from the basic psychological needs scale (Chen et al., 2015) was used to measure autonomy (4 items), competence (4 items) and relatedness (4 items). The sample titles are “I feel that I have the right to choose whether to participate in pickleball” (autonomy), “I feel capable when playing pickleball” (sense of competence), “I feel close to my partner with pickleball” (relatedness). The Cronbach’s alpha of the combined scale was 0.975 (subdimensions: autonomy 0.941, competence 0.947, relatedness 0.957).

Data collection of social media uses a 5-question scale adapted from the sports media use scale (Billings et al., 2017) to measure active participation in pickleball related content on TikTok, Xiaohongshu and WeChat platforms. The sample title is “I often watch pickleball videos on social media.” Cronbach’s alpha is 0.939.

The 18-item scale adapted from the physical activity motivation scale (Frederick and Ryan, 1993) was used for participation motivation, covering intrinsic motivation (6 items), extrinsic motivation (6 items) and social motivation (6 items). Cronbach’s alpha is 0.982.

Behavioral intention was assessed by using the 10-item scale adapted from the intention scale (Hagger et al., 2005) to assess the intention, planning, and willingness to participate in pickleball. Cronbach’s alpha is 0.971.

### 4.3 Data analysis

All analyses were performed using SPSS 27.0 and Mplus 8.3 with maximum-likelihood (ML) estimation. The analysis strategy proceeded in six stages. First, descriptive statistics and internal-consistency reliabilities were computed for all variables; eleven isolated missing item cells (<0.01% of all responses) were replaced by the rounded item mean, and composite scores were computed as available-item means. Second, the measurement model was estimated: a confirmatory factor analysis (CFA) with four correlated first-order factors and 45 indicators, followed by evaluation of convergent validity (average variance extracted, AVE ≥ 0.50; composite reliability, CR ≥ 0.70; Fornell and Larcker, 1981) and discriminant validity (heterotrait–monotrait ratio of correlations, HTMT, evaluated against the 0.85 and 0.90 criteria; Henseler et al., 2015). Third, common method bias (CMB) was assessed with three complementary diagnostics: Harman’s single-factor test, an unmeasured common latent factor model (Podsakoff et al., 2003; Liang et al., 2007), and full collinearity variance inflation factors (Kock, 2015). Fourth, Pearson correlations among the composite scores were inspected. Fifth, following the two-step logic of estimating the measurement model before the structural model, the structural model was estimated with all hypothesized paths entered simultaneously; model fit was evaluated with the chi-square statistic, *χ*^2^/d*f*, the comparative fit index (CFI), the Tucker–Lewis index (TLI), the root mean square error of approximation (RMSEA), and the standardized root mean square residual (SRMR), against conventional criteria (Hu and Bentler, 1999). Sixth, the indirect effects (H7–H9) were tested with bootstrap mediation analysis using 5,000 resamples and bias-corrected (BC) 95% confidence intervals (Hayes, 2018). Because resampling the 45-indicator latent model 5,000 times was computationally prohibitive, bootstrap standard errors and confidence intervals were computed on the parceled (composite-score) path model; point estimates from the latent structural model differed from the corresponding parceled estimates by less than 0.03 in absolute value. Statistical significance was evaluated at *α* = 0.05, and all path language in this paper is used in the statistical, non-causal sense.

## 5 Research results

### 5.1 Descriptive statistics

Table 1 shows the distribution of demographic characteristics. The sample was mainly male (66.0%) and undergraduate (64.4%). It is worth noting that 77.2% of the participants reported no pickleball experience, indicating that the sample captured individuals in the pre-exercise stage rather than established participants. This distribution enhances the practical relevance of the recruitment-oriented interventions found in this paper.

**Table 1:** Demographic characteristics, *N* = 1,992.

Category	Group	*n*	%	Cum. %
Gender	Male	1,314	66.0	66.0
	Female	678	34.0	100.0
Education	Undergraduate	1,282	64.4	64.4
	Junior College	709	35.6	99.9
	Other	1	0.1	100.0
Experience	None	1,537	77.2	77.2
	<1 yr	366	18.4	95.6
	1–3 yrs	89	4.5	100.0

**Figure 2:**
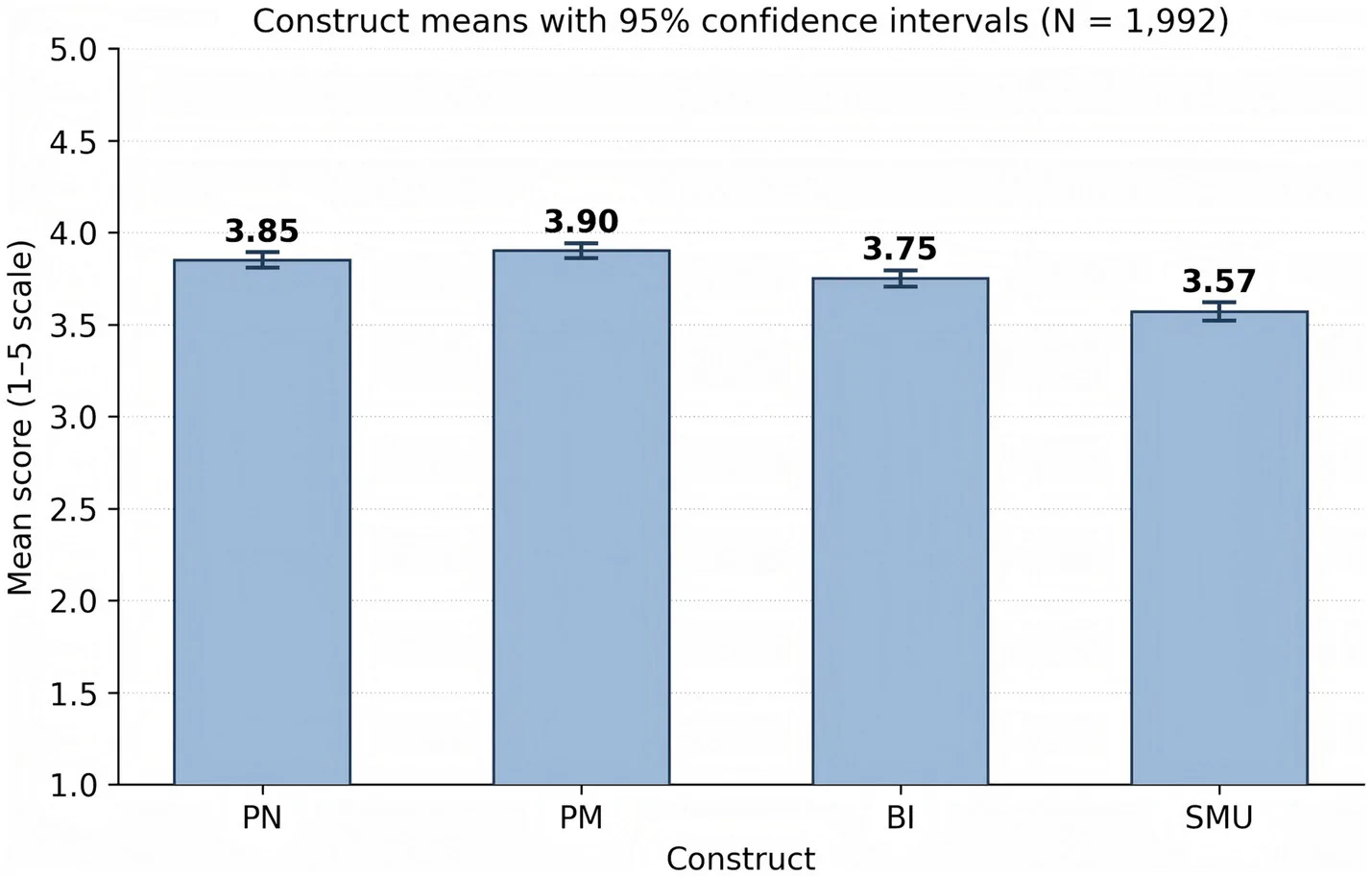
Mean scores across measured dimensions. Error bars represent 95% confidence intervals.

Figure 2 reports the descriptive statistics of the four main variables. Construct means ranged from 3.57 (social media use, SD = 1.11) to 3.90 (participation motivation, SD = 0.94), with psychological needs at 3.85 (SD = 0.96) and behavioral intention at 3.75 (SD = 1.02), indicating moderately positive scores on each construct. Cronbach’s *α* coefficients were 0.975 (PN), 0.939 (SMU), 0.982 (PM), and 0.971 (BI), indicating excellent internal consistency; alpha values above 0.95 may, however, also indicate item redundancy, a point taken up in the Limitations. Skewness (from −0.340 to −0.688) and excess kurtosis (from −0.619 to 0.264) were within conventional bounds, supporting the normality assumption of maximum-likelihood estimation.

### 5.2 Measurement model

The four-factor measurement model (45 indicators, ML estimation) showed mixed but acceptable fit: *χ*^2^ (939) = 14841.60, *p* < 0.001; *χ*^2^/d*f* = 15.81; CFI = 0.886; TLI = 0.879; RMSEA = 0.086, 90% CI [0.085, 0.087]; SRMR = 0.039. The absolute-fit indices (RMSEA, SRMR) were acceptable, whereas the incremental indices (CFI, TLI) fell slightly below the conventional 0.90 guideline—a pattern frequently observed in models with many indicators estimated on large samples, and acknowledged as a qualification in the Limitations. All standardized loadings were substantial and significant (*p* < 0.001): 0.819–0.902 for PN, 0.851–0.890 for PM, 0.853–0.910 for BI, and 0.810–0.894 for SMU. Convergent validity was excellent: the average variance extracted (AVE) exceeded 0.75 and composite reliability (CR) exceeded 0.93 for every construct (Table 2).

**Table 2:** Measurement model results: standardized loadings, convergent validity, and reliability.

Construct	Items	Std. loadings (range)	AVE	CR	Cronbach’s *α*
Psychological needs (PN)	12	0.819–0.902	0.766	0.975	0.975
Participation motivation (PM)	18	0.851–0.890	0.755	0.982	0.982
Behavioral intention (BI)	10	0.853–0.910	0.773	0.971	0.971
Social media use (SMU)	5	0.810–0.894	0.757	0.939	0.939

All standardized loadings significant at *p* < 0.001. Model fit: *χ*^2^ (939) = 14841.60, *p* < 0.001; *χ*^2^/d*f* = 15.81; CFI = 0.886; TLI = 0.879; RMSEA = 0.086, 90% CI [0.085, 0.087]; SRMR = 0.039. AVE, average variance extracted; CR, composite reliability.

### 5.3 Discriminant validity

Discriminant validity was assessed with the heterotrait–monotrait ratio of correlations (HTMT) and the Fornell–Larcker criterion (Table 3). All HTMT values were below the liberal 0.90 threshold; however, the HTMT for the PN–PM pair (0.879) exceeded the strict 0.85 criterion (Henseler et al., 2015). Consistently, the Fornell–Larcker criterion was met for all construct pairs except PN–PM, for which the latent correlation (0.881) marginally exceeded the square roots of the AVEs (0.875 for PN and 0.869 for PM). All other pairs passed both criteria. This pattern indicates that psychological needs and participation motivation, although conceptually distinct, are empirically close in the present data. The overlap is theoretically intelligible: within SDT’s motivational sequence, need satisfaction and autonomous motivation are adjacent, mutually reinforcing stages, so high empirical overlap is to be expected (Hagger et al., 2006). We nevertheless flag this marginal failure as a limitation and call for refined, more sharply differentiated measures in future research.

**Table 3:** Correlations and discriminant validity among constructs.

Construct	PN	PM	BI	SMU
PN	**0.875**	0.879	0.768	0.601
PM	0.881	**0.869**	0.806	0.645
BI	0.767	0.800	**0.879**	0.775
SMU	0.596	0.631	0.765	**0.870**

Diagonal (bold): square root of the AVE. Below diagonal: latent correlations (all *p* < 0.001). Above diagonal: HTMT ratios. Composite-score correlations (*N* = 1,992): PN–PM = 0.861, PN–BI = 0.748, PN–SMU = 0.574, PM–BI = 0.788, PM–SMU = 0.618, BI–SMU = 0.739 (all *p* < 0.001).

Two sensitivity tests corroborated the statistical distinctness of PN and PM despite their high correlation. First, chi-square difference tests strongly rejected the restricted models: merging PN and PM into a single factor substantially degraded fit (*χ*^2^ = 21044.25, d*f* = 942; Δ*χ*^2^ (3) = 6202.64, *p* < 0.001), as did constraining the PN–PM latent correlation to unity (Δ*χ*^2^ (1) = 6202.64, p < 0.001); the four-factor solution was retained in both comparisons. Second, a bootstrap analysis of the HTMT ratio (2,000 resamples) yielded a 95% confidence interval of [0.860, 0.897] for the PN–PM pair, which excludes both 0.90 and 1.00; by the inference criterion of Henseler et al. (2015), the two constructs are statistically distinguishable, although nearly the entire sampling distribution lies above the strict 0.85 benchmark. We therefore retain PN and PM as separate constructs while treating their empirical closeness as a substantive qualification (see Sections 5.4 and 6.4).

### 5.4 Common method bias

Three complementary diagnostics were used to evaluate common method bias (Table 4). First, Harman’s single-factor test showed that the first unrotated factor accounted for 65.3% of the variance under principal component analysis and 74.0% under principal axis factoring—both above the 50% criterion. Second, adding an orthogonal unmeasured common latent factor (Podsakoff et al., 2003; Liang et al., 2007) improved fit relative to the baseline four-factor model (*χ*^2^ = 11331.83, d*f* = 894; CFI = 0.914; TLI = 0.905; RMSEA = 0.077; ΔCFI = +0.029; ΔRMSEA = −0.010), and the method factor carried a mean standardized loading of 0.761, with its squared value accounting for approximately 59% of item variance on average and substantially shrinking the substantive loadings (by 0.61 on average). Third, full collinearity VIFs (Kock, 2015) exceeded the 3.3 criterion for PN (4.054), PM (4.740), and BI (3.762), but not for SMU (2.219).

**Table 4:** Common method bias diagnostics.

Diagnostic	Statistic	Result	Criterion	Evaluation
Harman single factor (PCA)	First-factor variance	65.3%	<50%	Exceeded
Harman single factor (PAF)	First-factor variance	74.0%	<50%	Exceeded
Common latent factor	Mean std. method loading	0.761 (≈ 59% of item variance)	—	Substantial
Common latent factor	Fit change vs. baseline	ΔCFI = +0.029; ΔRMSEA = −0.010	—	Fit improves
Full collinearity VIF	PN/PM/BI/SMU	4.054/4.740/3.762/2.219	<3.3	3 of 4 exceed

PCA, principal component analysis; PAF, principal axis factoring; VIF, variance inflation factor. Baseline four-factor model: *χ*^2^ (939) = 14841.60, CFI = 0.886, TLI = 0.879, RMSEA = 0.086; method-factor model: *χ*^2^ (894) = 11331.83, CFI = 0.914, TLI = 0.905, RMSEA = 0.077. Adding the method factor reduced the substantive standardized loadings by 0.61 on average.

Taken together, the three diagnostics converge: common method variance cannot be ruled out and is likely substantial, despite the procedural remedies in place (anonymity, confidentiality assurances, and attention checks; see Section 4.1). Consequently, all parameter estimates reported below should be read with this qualification, and we return to its implications in the Discussion.

A further robustness check re-estimated the structural model with the orthogonal method factor partialled. Under this specification, the paths involving participation motivation changed markedly: the PN → PM path was no longer significant (*β* = 0.039, *p* = 0.440), and the PM → BI and SMU → PM paths reversed sign (*β* = −0.357 and *β* = −0.346, respectively), whereas the PN → BI (*β* = 0.216) and SMU → BI (*β* = 0.433) paths were essentially unchanged. We interpret this pattern with caution: because need satisfaction and participation motivation are conceptually adjacent stages of the same motivational sequence, an orthogonal factor that removes all variance shared across constructs inevitably removes genuine substantive covariance as well, and sign reversals of this kind are a recognized symptom of over-partialling in common-latent-factor models (Podsakoff et al., 2003). The check nonetheless shows that the central mechanism of the model cannot be statistically separated from the general evaluative factor in single-source self-report data. We therefore treat the CLF-adjusted estimates as a diagnostic check rather than as a corrected model; the primary mediation results reported below are presented as theoretically ordered associations, with the understanding that their magnitudes are likely inflated by shared method variance. Multi-method replication is accordingly identified as an essential next step (Section 6.4).

### 5.5 Correlation analysis

**Figure 3:**
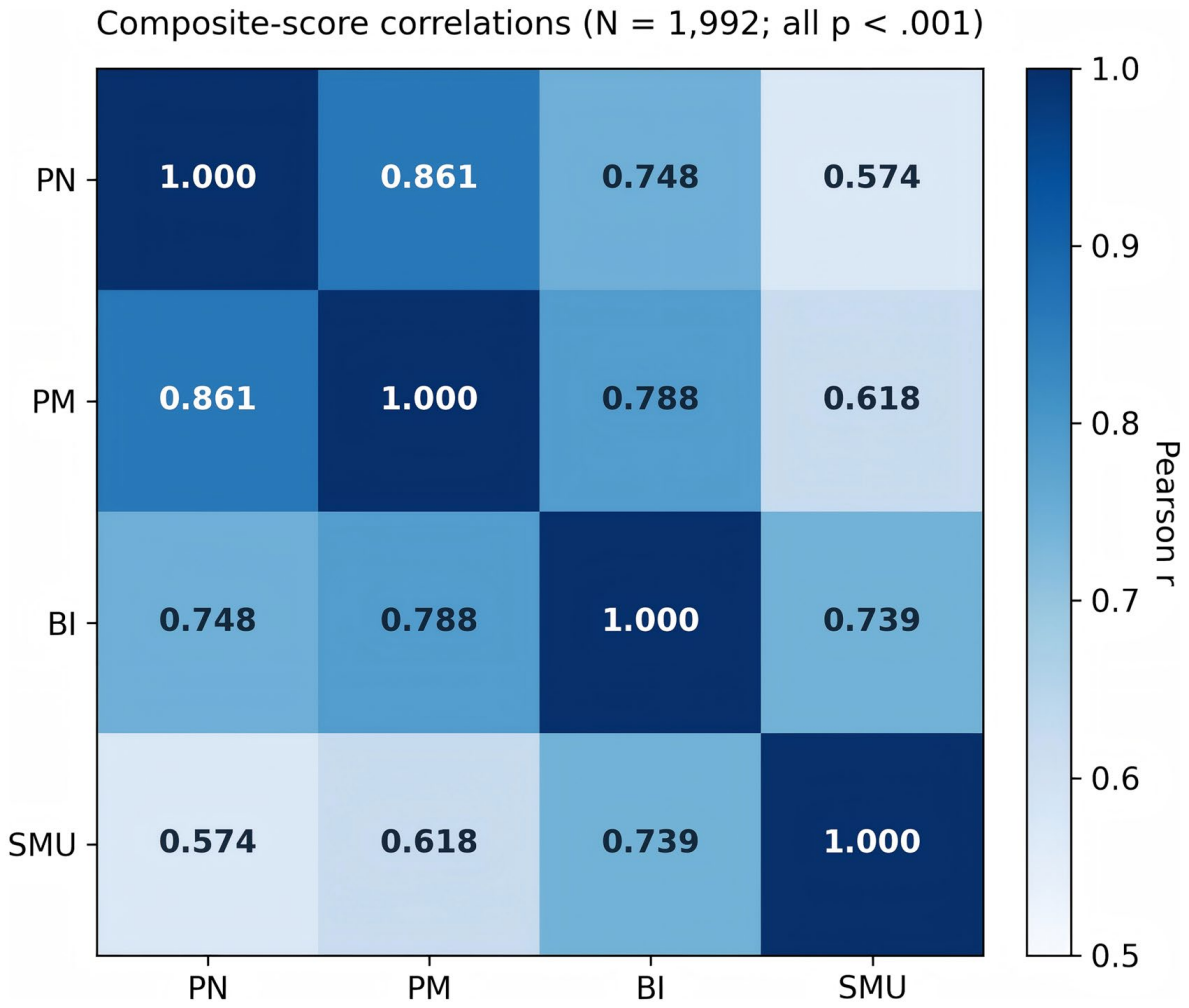
Correlation heatmap. Darker colors indicate stronger positive correlations.

Figure 3 shows the correlation matrix between variables. There was a significant positive correlation between all variables (*p* < 0.001). The correlation between psychological needs and participation motivation was the strongest (*r* = 0.861), followed by behavioral intention (*r* = 0.748). The relevant models meet the theoretical expectations and provide preliminary support for the hypothetical path.

### 5.6 Structural model and direct effects

**Figure 4:**
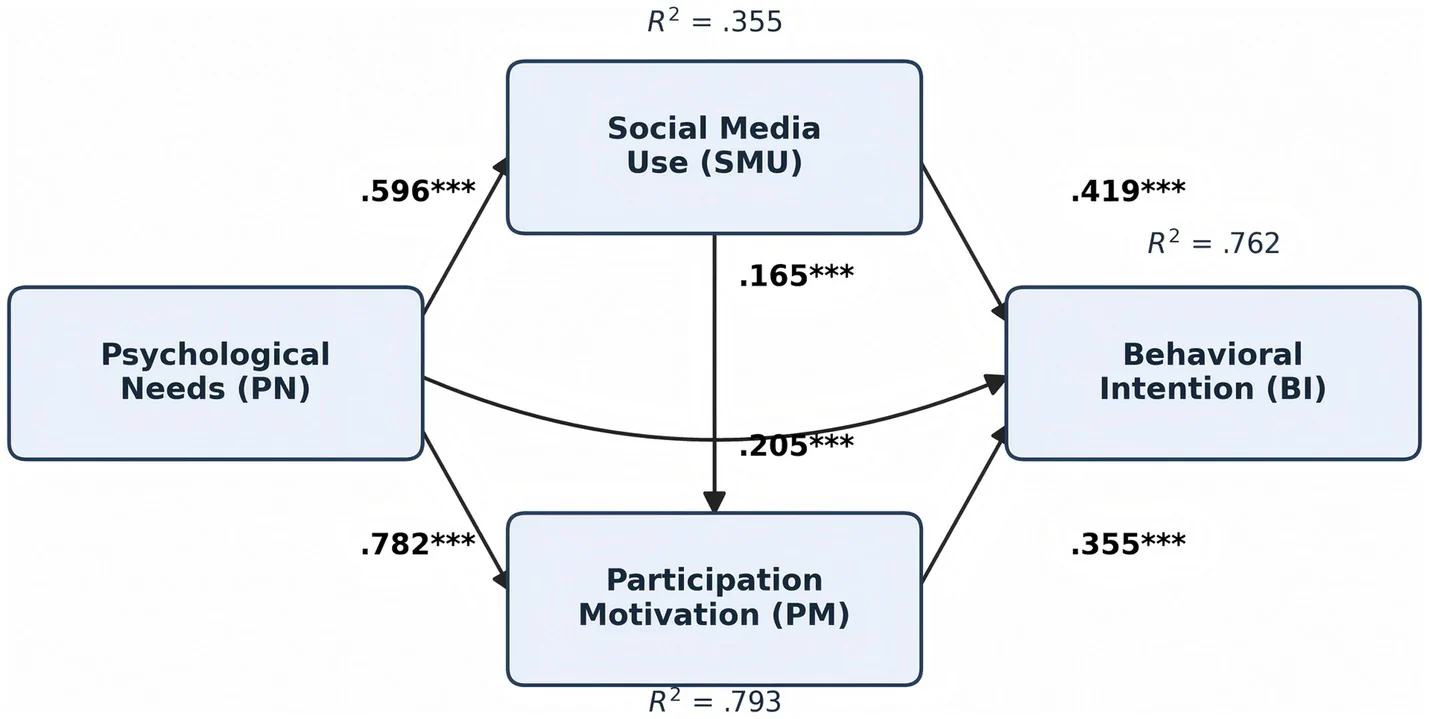
Standardized path coefficients of the simultaneous structural model. ***p < 0.001.

Because the structural part of the model is saturated (just-identified), the fit of the simultaneous structural model is identical to that of the measurement model: *χ*^2^ (939) = 14841.60, *p* < 0.001; *χ*^2^/d*f* = 15.81; CFI = 0.886; TLI = 0.879; RMSEA = 0.086, 90% CI [0.085, 0.087]; SRMR = 0.039. Table 5 reports the standardized direct paths estimated with all hypothesized effects entered simultaneously. All six paths were positive and significant (*p* < 0.001), supporting H1–H6 (Figure 4). Psychological needs were associated with behavioral intention (*β* = 0.205, SE = 0.030), social media use (*β* = 0.596, SE = 0.024), and participation motivation (*β* = 0.782, SE = 0.022). Social media use (*β* = 0.419, SE = 0.019) and participation motivation (*β* = 0.355, SE = 0.030) were both associated with behavioral intention, and social media use was additionally associated with participation motivation (*β* = 0.165, SE = 0.015).

**Table 5:** Standardized direct paths of the simultaneous structural model.

Hypothesis	Path	*β*	SE	*p*	Result
H1	PN → BI	0.205	0.030	<0.001	Supported
H2	SMU → BI	0.419	0.019	<0.001	Supported
H3	PN → SMU	0.596	0.024	<0.001	Supported
H4	PM → BI	0.355	0.030	<0.001	Supported
H5	PN → PM	0.782	0.022	<0.001	Supported
H6	SMU → PM	0.165	0.015	<0.001	Supported

Simultaneous latent structural equation model; standardized coefficients. The structural part is saturated, so model fit equals the measurement-model fit (see Table 2). *R*^2^: SMU = 0.355; PM = 0.793; BI = 0.762. PN, psychological needs; SMU, social media use; PM, participation motivation; BI, behavioral intention.

The model accounted for 35.5% of the variance in social media use, 79.3% of the variance in participation motivation, and 76.2% of the variance in behavioral intention (*R*^2^ = 0.355, 0.793, and 0.762, respectively). The explained variance for behavioral intention is high relative to typical intention models; given the common-method findings in Section 5.4 and the empirical closeness of the PN and PM constructs (Section 5.3), part of this explanatory power is likely attributable to shared method variance and construct overlap rather than to substantive association alone—an interpretation developed further in the Discussion.

A parcel-level re-specification of the structural model (PN: three subdimension parcels; PM and BI: three sequential parcels each; SMU: the five items; 14 indicators in total) yielded improved fit (*χ*^2^ = 1529.07, d*f* = 71; CFI = 0.959; TLI = 0.948; RMSEA = 0.102; SRMR = 0.039) and closely similar standardized paths: PN → BI 0.216, SMU → BI 0.432, PN → SMU 0.603, PM → BI 0.334, PN → PM 0.808, and SMU → PM 0.140 (all *p* < 0.001), with *R*^2^ = 0.363, 0.810, and 0.767 for SMU, PM, and BI, respectively. All six direct hypotheses were therefore robust to measurement specification.

### 5.7 Mediation analysis

Table 6 presents the bootstrap mediation results (5,000 resamples, bias-corrected 95% confidence intervals, computed on the parceled path model; see Section 4.3). All three indirect effects were significant: via social media use, IE = 0.226, 95% BC CI [0.192, 0.263] (H7); via participation motivation, IE = 0.279, 95% BC CI [0.215, 0.346] (H8); and via the sequential chain, IE = 0.039, 95% BC CI [0.027, 0.055] (H9). The total indirect effect was 0.544 [0.467, 0.613]; the direct effect of psychological needs on behavioral intention was 0.204 [0.125, 0.293]; and the total effect was 0.748 [0.709, 0.783]. Because all confidence intervals exclude zero, H7–H9 are supported.

**Table 6:** Bootstrap mediation analysis results (5,000 resamples).

Effect	Estimate	95% BC CI	% of total effect	% of total indirect
H7: PN → SMU → BI	0.226	[0.192, 0.263]	30.2%	41.6%
H8: PN → PM → BI	0.279	[0.215, 0.346]	37.3%	51.2%
H9: PN → SMU → PM → BI	0.039	[0.027, 0.055]	5.2%	7.2%
Total indirect effect	0.544	[0.467, 0.613]	72.7%	100%
Direct effect (PN → BI)	0.204	[0.125, 0.293]	27.3%	—
Total effect (PN → BI)	0.748	[0.709, 0.783]	100%	—

IE, indirect effect; BC CI, bias-corrected confidence interval. Standardized effects estimated on the parceled path model (see Section 4.3); latent-SEM point estimates differed by less than 0.03. All CIs exclude zero.

**Figure 5:**
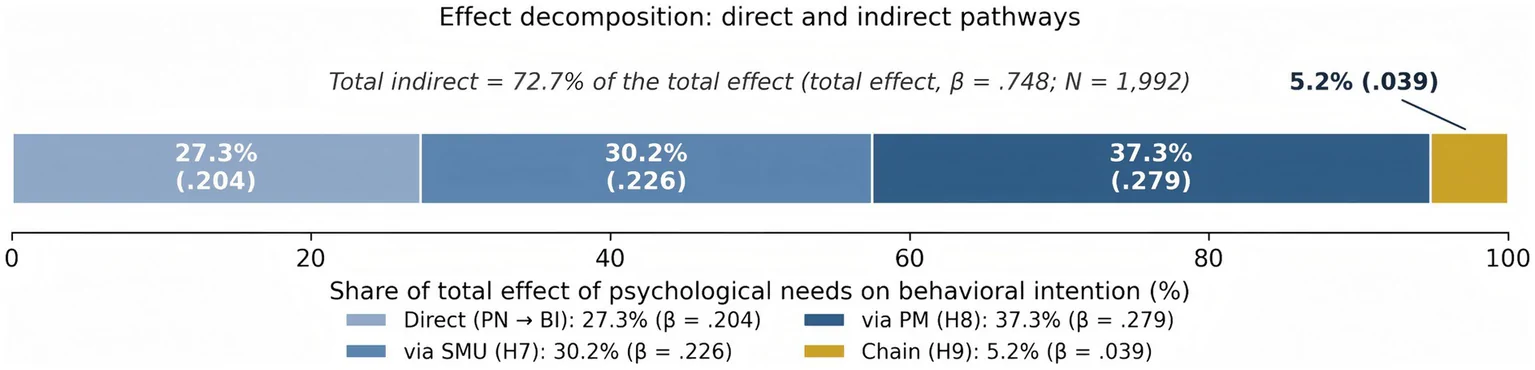
Effect decomposition of the total effect of psychological needs on behavioral intention: direct path, 27.3%; indirect via social media use (H7), 30.2%; via participation motivation (H8), 37.3%; via the sequential chain (H9), 5.2%.

Decomposition of the total effect shows that 27.3% is carried by the direct path and 72.7% by the indirect paths combined (H7: 30.2%; H8: 37.3%; H9: 5.2%) (Figure 5). Of the total indirect effect, participation motivation carried the largest share (51.2%), followed by social media use (41.6%), with the chain path contributing 7.2%. Participation motivation thus remains the dominant mediator, while the chain path, although small, is statistically reliable.

The parcel-level model yielded consistent indirect effects (1,000 bias-corrected bootstrap resamples): H7 = 0.260, 95% CI [0.220, 0.303]; H8 = 0.270 [0.167, 0.370]; and the chain effect = 0.028 [0.014, 0.046], with a total indirect effect of 0.558 [0.446, 0.661] and a total effect of 0.774. H7–H9 were therefore supported under both measurement specifications. One qualification emerged: at the parcel level the two single-mediator effects were nearly equal, so the dominance of participation motivation observed in the primary analysis (51.2% of the total indirect effect) should be read as specification-dependent rather than definitive.

### 5.8 Summary of hypothesis testing

Table 7 offers a complete summary. All nine hypotheses were supported at *p* < 0.001—supported, that is, in the sense of statistical association, subject to the cross-sectional design and the common-method-variance qualification documented in Section 5.4. The model accounted for 76.2% of the variance in behavioral intention.

**Table 7:** Summary of hypothesis testing results.

Hypothesis	Content	Type	Estimate	Result
H1	PN → BI	Direct	*β* = 0.205***	Supported
H2	SMU → BI	Direct	*β* = 0.419***	Supported
H3	PN → SMU	Direct	*β* = 0.596***	Supported
H4	PM → BI	Direct	*β* = 0.355***	Supported
H5	PN → PM	Direct	*β* = 0.782***	Supported
H6	SMU → PM	Direct	*β* = 0.165***	Supported
H7	PN → SMU → BI	Indirect	IE = 0.226***	Supported
H8	PN → PM → BI	Indirect	IE = 0.279***	Supported
H9	PN → SMU → PM → BI	Indirect (chain)	IE = 0.039***	Supported

PN, psychological needs; SMU, social media use; PM, participation motivation; BI, behavioral intention; IE, indirect effect; ***p < 0.001.

## 6 Discussion

### 6.1 Main findings

The present study tested an integrated chain mediation model linking psychological needs, social media use, participation motivation, and behavioral intention in the context of college pickleball participation. All nine hypotheses were supported, and the model accounted for 76.2% of the variance in behavioral intention. Read together with the measurement and common-method diagnostics, the findings carry several theoretical and practical implications, which we discuss below in relation to prior research.

First, psychological needs showed the strongest single association in the model, predicting participation motivation at *β* = 0.782 and, jointly with social media use, accounting for 79.3% of its variance. This pattern is consistent with the motivational sequence at the heart of self-determination theory, in which need satisfaction energizes autonomous motivation (Hagger et al., 2006; Ryan et al., 2006), and it extends that sequence to an emerging leisure sport in a Chinese college sample (Abula et al., 2020). The magnitude of the association, however, exceeds the moderate coefficients typically reported in tests of this sequence (Hagger et al., 2006) and in need–motivation research on digital play (Ryan et al., 2006). Two design features plausibly inflate the estimate: all constructs were self-reported in a single survey, and the PN and PM measures were only marginally discriminable (HTMT = 0.879). The finding should therefore be read as strong but likely upward-biased support for the needs–motivation linkage.

Second, social media use emerged as the largest direct predictor of behavioral intention (*β* = 0.419), ahead of participation motivation (*β* = 0.355) and psychological needs (*β* = 0.205). This is broadly consistent with evidence that fitness-related social media use is associated with exercise intention and behavior among Chinese college students (Xiao et al., 2025) and with uses-and-gratifications accounts in which active, goal-directed media engagement supplies information, behavioral models, and social connection relevant to participation (Omar and Dequan, 2020). The comparatively strong direct association observed here may reflect our sport-specific operationalization of media use—active engagement with pickleball content rather than general screen time—although shared method variance again cautions against over-interpretation. Consistent with this reading, college students appear to use social media platforms not merely as passive entertainment tools but as instruments for actively discovering, evaluating, and investing in sport participation opportunities (Johnston and Davis, 2019).

Third, participation motivation was the dominant mediator, carrying 51.2% of the total indirect effect (IE = 0.279). This echoes the trans-contextual model, in which autonomous motivation transmits the association of need-related experiences to intentional behavior (Hagger et al., 2003; Hagger and Chatzisarantis, 2016), and meta-analytic SDT–TPB integrations in which motivational variables sit between need constructs and intention (Hagger and Chatzisarantis, 2009). From the perspective of the theory of planned behavior, the results are consistent with the view that background factors are associated with intention chiefly through proximal motivational mechanisms. This ordering, however, was specification-dependent: in the parcel-level robustness check the two single-mediator paths were nearly equal (Section 5.7), so the dominance of participation motivation is best regarded as suggestive rather than definitive.

Fourth, the chain path—psychological needs → social media use → participation motivation → behavioral intention—was small but significant, accounting for 5.2% of the total effect (IE = 0.039 [0.027, 0.055]). This closely parallels the chained indirect share reported by Xiao et al. (2025) for fitness social media use (approximately 4.25% of the total effect), suggesting that sequential need–media–motivation processes replicate across sport-content domains. The modest magnitude is not unexpected: the translation of media engagement into enhanced motivation likely requires stronger contextual triggers (e.g., peer invitations, campus events) than the present model captures, and the two single-mediator pathways together carry the primary explanatory weight—a pattern consistent with prior mediation research in which correlated mediators exhibit asymmetric effect distributions.

Finally, the findings complement the emerging pickleball literature. Prior work has examined older adults’ well-being and serious leisure (Heo et al., 2018), the behavioral intention of existing players within a TPB framework (Wang et al., 2023), and the drivers of pickleball participation with mixed-methods designs (Huang et al., 2026). The present results add a need-based, media-sensitive account focused on Chinese college students, most of whom had no prior playing experience. At the same time, the explained variance in behavioral intention (76.2%) is considerably higher than the values typically reported in TPB-based intention models (Hagger et al., 2002a; Hagger and Hamilton, 2024); we attribute this partly to shared method variance and the conceptual overlap of adjacent constructs, and we treat it as a qualification on the findings rather than as a virtue of the model.

### 6.2 Theoretical contribution

This paper makes four contributions to the theoretical literature. The primary contribution lies in integrating self-determination theory, the theory of planned behavior, and uses and gratifications theory into a unified explanatory framework in which the three theories operate at complementary levels: a distal need engine, a media-channel mechanism, and a proximal intention-formation system. Prior integrations have joined two of these perspectives at a time (Hagger and Chatzisarantis, 2009; Sheldon et al., 2011; Gao et al., 2021); the present results, in which all direct and indirect paths were simultaneously significant, provide initial evidence that the three-way integration is empirically coherent rather than merely additive. These theories are thus best read not as competing explanations but as complementary lenses on the formation of participation intention.

The second contribution involves extending self-determination theory to an emerging leisure sport. SDT’s evidence base in exercise science rests largely on established activities (Teixeira et al., 2012); the significant need-related paths observed here are consistent with the theory’s core propositions traveling to novel sport contexts and to the pre-participation stage, indicating that need–behavior linkages are not bound to specific activity types.

The third contribution relates to the conceptualization of social media use in sport participation research. Rather than treating media consumption as a general behavioral category, we operationalized it as active, sport-specific engagement, in line with UGT’s emphasis on goal-directed media selection (Katz et al., 1974; Omar and Dequan, 2020; Meng and Leung, 2021). The finding that social media use was the largest direct predictor of behavioral intention suggests that this specificity is consequential and provides a template for future sport media research.

The fourth contribution is documenting the need–media–motivation chain in a large Chinese college sample, thereby extending need–media findings from general social platforms (Gao et al., 2021) and fitness-content research (Xiao et al., 2025) to an emerging racket sport. The replication of a small but significant chain effect across these contexts strengthens the case that sequential mediation is a general feature of digitally mediated sport participation rather than an idiosyncrasy of a single platform or activity.

### 6.3 Practical implications

The findings have practical implications for multiple stakeholder groups. For campus sports managers, the dominant position of psychological needs indicates that the recruitment information should emphasize how pickleball meets autonomy (form selection, flexible arrangement), competence (skills advancement, mastery opportunities), and relatedness (doubles, social networking, community construction). Communication based on demand framework may be more effective than purely informational or promotional methods.

For sports marketers and social media managers, the significant direct and intermediary effects of social media use highlight the strategic importance of the platform. Creating and disseminating content that visually shows the attributes that pickleball needs to meet, such as friendship and skill development tracks between participants, may help translate need-related interests into participation motivation and behavioral intention. Given the media consumption habits of college students, short video content that shows the experience of real participants may be particularly effective.

For policy makers who pay attention to adolescent sports activities, the model is consistent with the possibility that interventions supporting psychological needs in educational contexts are associated with downstream participation outcomes through two pathways: media engagement and motivation. Integrating demand supporting elements into physical education curriculum may be a cost-effective upstream strategy.

### 6.4 Limitations and future directions

There are some limitations in this paper. First, the cross-sectional design precludes causal and temporal inference: although the theoretical model specifies directional paths, the data cannot establish temporal priority, and the estimated paths should be read as theoretically ordered associations only. Longitudinal or experimental designs will be required to strengthen any causal claims. We chose the cross-sectional method mainly for practical reasons, including resource constraints and the exploratory nature of this paper.

Second, the exclusively self-report measurement invites common method bias (CMB), and the three diagnostics reported in Section 5.4 converge in indicating that it is substantial: the first unrotated factor accounted for 65.3% of the variance, an orthogonal method factor absorbed roughly 59% of the average item variance while improving model fit, and full collinearity VIFs exceeded 3.3 for three of the four constructs. Although procedural remedies were in place (anonymity and confidentiality assurances, attention checks), the parameter estimates—including the *R*^2^ of 0.762 for behavioral intention—are likely inflated by shared method variance. Moreover, when the structural paths were re-estimated with the method factor partialled, the paths involving participation motivation became non-significant or reversed sign (Section 5.4), indicating that the mediating mechanism cannot be separated from the general evaluative factor with single-source self-report data; the structural results are therefore presented as theoretically ordered associations only. Future research should separate measurement occasions temporally, incorporate objective or multi-source measures (e.g., actual participation frequency tracked by venue check-in or wearable devices), and include theoretically unrelated marker variables to partial out method effects (Podsakoff et al., 2003). Relatedly, post-hoc inspection revealed that approximately 14% of respondents selected the highest scale anchor on every item of the questionnaire (16% when only the 45 analysis items are considered), a response pattern that may reflect acquiescence or socially desirable responding. Although this pattern is partly captured by the common-method diagnostics already reported (Section 5.4), future research with balanced scales or instructed-response items would help disentangle substantive variance from response-style variance.

Third, discriminant validity between psychological needs and participation motivation was only marginally supported (HTMT = 0.879; the Fornell–Larcker criterion failed for the PN–PM pair). Chi-square difference tests and HTMT bootstrap inference supported the statistical distinctness of the two constructs (Section 5.3), but they remain empirically very close. Although the two constructs are adjacent stages in SDT’s motivational sequence, future work should sharpen their empirical separation—for example, by distinguishing need satisfaction from need frustration (Chen et al., 2015) and by using behaviorally anchored motivation items.

Fourth, the alpha coefficients were very high (0.939–0.982), which may indicate item redundancy and unnecessarily lengthen the instrument; validated short forms would reduce respondent burden in replications.

Fifth, the incremental fit indices fell below conventional thresholds (CFI = 0.886, TLI = 0.879), a pattern that is common in many-indicator models estimated on large samples but that nonetheless qualifies the adequacy of the measurement model.

Sixth, the sample was recruited by convenience, was majority male, and comprised 77.2% of respondents without pickleball experience. The model therefore speaks to intention formation in a largely pre-participation population, and the intention–behavior gap remains unaddressed; generalization to female students, graduate students, established players, and universities with different levels of infrastructure requires further research.

Seventh, the measurement of social media use in this paper focuses on active participation in pickleball content, rather than passive exposure or general platform use. Future research can use empirical sampling method to capture real-time media consumption and its immediate motivational consequences.

## 7 Conclusion

We proposed and tested an integrated chain mediation model of the psychological and digital pathways associated with college students’ intention to participate in pickleball. Drawing on self-determination theory, the theory of planned behavior, and uses and gratifications theory, the study found that psychological needs were associated with behavioral intention both directly (*β* = 0.205) and through three indirect channels—social media use, participation motivation, and their sequential combination—with all nine hypothesized paths supported in the statistical sense.

The model accounted for 76.2% of the variance in behavioral intention. Participation motivation carried the largest share of the total indirect effect in the primary specification (51.2%), although the two single-mediator paths were nearly equal in the parcel-level robustness check; social media use contributed significantly to both direct and indirect associations, and the chain mediation path, although modest (5.2% of the total effect), was statistically reliable and consistent with the sequential character of the hypothesized psychological process. It should be noted, however, that all three common-method diagnostics indicated substantial method variance; such high explanatory power may be partly attributable to the use of exclusively self-report data and conceptually overlapping constructs (Podsakoff et al., 2003). Future research should replicate the model with longitudinal designs and objective behavioral measures to further validate these findings.

These findings advance the theoretical understanding of how sport participation intentions are formed and provide evidence-informed guidance for stakeholders seeking to promote emerging leisure sports among young adults. As pickleball continues its global expansion, understanding the psychological and digital mechanisms associated with participation intentions will be increasingly critical for effective recruitment, retention, and program development.

## Data Availability

The datasets presented in this article are not readily available because the dataset is not publicly available due to participant privacy/ethical restrictions/proprietary reasons. Anonymized data may be available from the corresponding author upon reasonable request, pending ethical approval. Requests to access the datasets should be directed to Jianfeng Hu, huguess211@hotmail.com.
